# A rare case report of pyogenic hepatic abscess secondary to sigmoid diverticulitis

**DOI:** 10.1177/2050313X261449110

**Published:** 2026-05-12

**Authors:** Hari Movva, Yasamin Rastgar, Arkoon Ali, Ethan Glass, Nghia Nguyen, Gurinder Luthra

**Affiliations:** 1 Department of Internal Medicine, University of Texas Medical Branch, Galveston, TX, USA; 2 School of Medicine, University of Texas Medical Branch, Galveston, TX, USA; 3 GastroDoxs, Houston, TX, USA; 4 Division of Gastroenterology, Division of Internal Medicine, University of Texas Medical Branch, Galveston, TX, USA

**Keywords:** diverticulitis, complication, hepatic abscess

## Abstract

Hepatic abscesses are uncommon and rarely associated with diverticulitis as an indirect source of infection. We report the case of a 60-year-old male with a history of recurrent diverticulitis who presented with fever, nausea, and abdominal pain demonstrating a single multiloculated hepatic lesion measuring 5.8 × 5.1 × 5.1 cm upon admission. Piperacillin–tazobactam was initiated for sepsis management, and metronidazole was initiated for amoebic coverage. Aspiration and drainage of the abscess were performed, and cultures confirmed *Streptococcus intermedius*, prompting antibiotic therapy to be narrowed to ceftriaxone and metronidazole. Subsequent imaging showed a reduction in the size of the abscess. A pyogenic liver abscess can rarely arise from hematogenous spread of infection via the portal venous system in the setting of diverticulitis. Early imaging, prompt antimicrobial therapy, and source control are essential to optimize outcomes and prevent complications.

## Introduction

Hepatic abscesses are relatively rare but clinically significant infections, with an incidence as low as 2.3 cases per 100,000 people and primarily occurring in males. Despite their rarity, it is associated with high mortality rates of up to 19% for in-hospital admissions.^1^ Common risk factors include proton pump inhibitor use, diabetes mellitus, prior liver surgery, and hepatic malignancy. Common sources are biliary tract disease, penetrating trauma, portal venous seeding from intra-abdominal infections, or hematogenous spread from infection sites.^1^

The microbiology of pyogenic liver abscesses (PLAs) is generally polymicrobial. Gram-positive organisms include *Streptococcus* and *Staphylococcus*, and gram-negative organisms include *Escherichia coli* and *Klebsiella pneumoniae.* In general, if gram-positive organisms are isolated, the diagnostic focus should include finding another source of infection that has hematogenous spread to the liver.^1^ Previously, *Streptococcus* was the most common bacterium for PLAs in North America, but this has been replaced by *Klebsiella.*^2^ *Klebsiella* is an unusual pathogen that is most common in gas-forming PLAs. The pathophysiology of increased gas production, impaired gas transportation, and impaired gas equilibrium is thought to coincide with diabetes mellitus. With this organism, liver enzymes are worse, and mortality rates are higher.^3^ Drug-resistant PLAs occur in gram-negative-heavy abscesses mostly containing *E. Coli, Klebsiella*, the *Pseudomonas* family, and *Enterobacter*, which make its treatment especially difficult.^4^ Anaerobic bacteria, particularly Bacteroides and Fusobacterium species, are rarer in PLAs and usually originate from the colon. Prognosis for these organisms is positive since they respond well to antibiotics, but isolating them from cultures is difficult, leading to underdiagnosis of anaerobes in PLAs.^5^ Fungal liver abscesses include those in the *Aspergillus* and *Candida* species that have been reported. In these cases, they are associated with higher morbidity due to delayed diagnosis and limited treatment options. They often occur in immunocompromised individuals and those with hematologic malignancies.^6,7^

The liver has a unique pathophysiology in which it receives blood circulation from the portal and systemic systems. In this way, it is more susceptible to disease. An intra-abdominal infection can lead to septic thrombophlebitis of the mesenteric and portal veins, known as pylephlebitis, leading to PLAs. The most common cause of pylephlebitis is diverticulitis and appendicitis.^8^ Many cases in the literature record the *Fusobacterium* species being implicated as the main pathogen, although this occurs in less than 5% of cases. Traditionally, it is known to cause Lemierre’s disease when an oral infection invades the internal jugular vein, leading to septic thrombophlebitis. In gastrointestinal cases, it is linked with gastrointestinal cancer and portal vein thrombosis.^9^

Clinically, PLA often presents with nonspecific symptoms, although fever is prevalent in 90% of cases. Other symptoms include night sweats, right upper quadrant pain, nausea, cough, dyspnea, malaise, and anorexia, making early diagnosis challenging.^1^

Diverticulitis is an inflammatory disease of diverticula and is a complication of diverticulosis. It more commonly occurs in older adults. Complications of diverticulitis include abscess, fistula, bowel obstruction, or perforation.^10^ Diverticulitis is thought to result in hepatic abscesses due to drainage via the portal venous route or when a rupture is present.^11^ Sigmoid diverticulitis as a source of a PLA is a rare cause.

In this report, we present a rare case of a PLA secondary to sigmoid diverticulitis caused by *Streptococcus intermedius*. This case highlights an unusual but important complication of diverticulitis and underscores the need for early recognition, appropriate imaging, and targeted antimicrobial therapy.

## Case presentation

A 60-year-old male with a past medical history of hypertension, hyperlipidemia, gastroesophageal reflux disease, and moderate to severe diverticulosis complicated by recurrent diverticulitis presented to the emergency department with 3 days of fever, nausea, and abdominal pain. The patient reported pain in his epigastric region and right upper quadrant, with associated nausea but no vomiting, and a recorded home temperature peaking at 104.5ºF. Vitals on arrival demonstrated tachycardia to 120 beats per min, blood pressure of 97/64, and a temperature of 102ºF. Physical exam demonstrated right upper quadrant tenderness without peritoneal signs. Further history was insignificant, with no prior abdominal surgeries. Routine labs were significant for leukocytosis, elevated liver enzymes, and hyperbilirubinemia, as shown in Table 1.

**Table 1.** Laboratory investigations.

Blood test	Observed value	Reference range	units
White cell count	14.12	4.0–11.0	×10^9^/L
AST	76	12–38	U/L
ALT	88	10–40	U/L
Total bilirubin	1.4	0.1–1.0	mg/dL

AST: aspartate aminotransferase; ALT: alanine aminotransferase.

**Figure 1.**
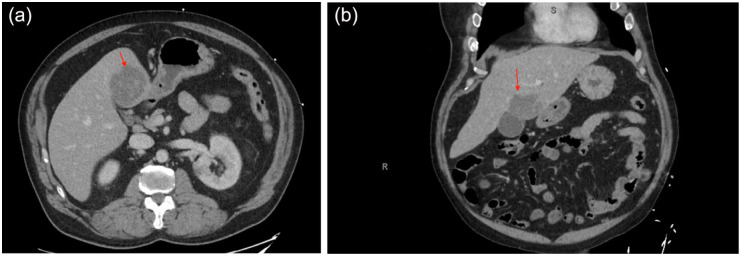
CTAP with contrast. (a) Axial view of hepatic abscess (red arrow). (b) Coronal view of hepatic abscess (red arrow). CTAP: computed tomography of the abdomen and pelvis.

Imaging of the abdomen was obtained via computed tomography (CT) of the abdomen and pelvis (CTAP) with contrast and noted low-grade acute diverticulitis of the sigmoid colon and a single, new liver lesion measuring 5.8 × 5.1 × 5.1 cm with concern for possible abscess versus primary malignancy (Figure 1). Blood cultures were obtained, and the patient was admitted with concerns for sepsis and started on piperacillin–tazobactam (Zosyn) 4.5 g intravenous every 8 h. Magnetic resonance imaging confirmed a 6 cm multiloculated hepatic abscess. Malignancy markers (CEA, AFP, CA 19–9) were within normal limits. Interventional radiology placed a drain and aspirated a substance resembling “anchovy paste” from the abscess. Infectious disease was consulted the same day and recommended metronidazole 750 mg intravenously three times a day for amoebic coverage based on aspirate characteristics, along with pyogenic coverage with Zosyn. Differential diagnosis leaned toward *Entamoeba histolytica* as the likely pathogen, and Zosyn was replaced with cefepime 2 g IV every 8 h to cover for a pyogenic cause. The patient showed clinical improvement, with a reduction in fevers and abdominal pain. Approximately 1 week after admission, the culture grew *Streptococcus intermedius*, and cefepime was replaced with ceftriaxone 2 g intravenous every 24 h alongside metronidazole. Blood cultures showed no isolated organism, and amoebic serology was negative. The patient was transitioned to oral antibiotics with plans to start amoxicillin–clavulanate 875/125 mg twice daily for a 2–4 week course. A transthoracic echocardiogram was recommended for bacteremia seeding from the liver, which was unremarkable. Repeat CTAP demonstrated a decrease in abscess size to 5 × 4.4 × 3.3 cm. The patient followed up with outpatient with resolution of symptoms on Augmentin with an appointment for an outpatient colonoscopy.

## Discussion

PLA is a rare but serious infection. It commonly arises from biliary tract disease, but gastrointestinal sources such as diverticulitis are an uncommon etiology. The proposed mechanism involves bacteria being spread from the bowel to the portal vein, resulting in pylephlebitis and abscess formation.^12^ The liver was likely seeded via hematogenous spread by the inferior mesenteric vein from the sigmoid colon in this patient. Our case highlights this uncommon pathway, in which the patient had a risk factor for diverticulitis.

Diverticulosis and diverticulitis are common conditions. The majority of patients with diverticulitis have uncomplicated disease; however, 10% will develop complications, which may include perforation, abscess, bowel obstruction, bleeding, or fistulas.^13^ Episodes of diverticulitis preceding or causing a liver abscess are a rare occurrence. A case series published by Kubovy et al.^14^ demonstrated preexisting diverticulosis or diverticulitis in 12% of PLA cases.

Our patient met sepsis criteria based on his fever, tachycardia, and leukocytosis. Prompt initiation of broad-spectrum antimicrobial therapy was started, and, along with percutaneous drainage, likely contributed to the favorable outcome.

Bacterial liver abscesses are often polymicrobial.^1^ A 2018 study by Wang et al. characterized the bacterial makeup of 178 patients with liver abscesses and identified 102 strains. These strains included *Klebsiella pneumoniae* (80.3%), gram-positive cocci (8.8%), *Escherichia coli* (7.8%), and *Pseudomonas aeruginosa* and *Acinetobacter baumannii* at low percentages.^7^ Previous population-based studies had demonstrated *Streptococcus* and *Escherichia coli* species to be the most prevalent, but recent data have shown a rise in *Klebsiella*-based abscesses.^15^ By contrast, fungal liver abscesses, most commonly caused by *Aspergillus* species, are rare and typically occur in immunocompromised hosts, such as those with hematologic malignancies, organ transplantation, or prolonged neutropenia. Compared to bacterial abscesses, fungal abscesses are associated with significantly worse outcomes due to delayed diagnosis and limited therapeutic options.^7^ By contrast, our patient was immunocompetent, presented with acute systemic symptoms, and demonstrated rapid clinical improvement with targeted antibacterial therapy, supporting a bacterial etiology.

The isolate in our patient was a member of the *Streptococcus* family. *Streptococcus intermedius* is a gram-positive cocci known to cause infections such as meningitis, endocarditis, and abscesses.^16^ Several case reports are useful comparisons to our patient. *Streptococcus intermedius* was the causative pathogen, although blood cultures were able to identify this, whereas our patient was diagnosed on aspirate culture. Imaging in our case allowed initiation of microbial therapy, where comparatively malignancy was more so ruled in Muscat.^16^ Previous literature has also cited the *Streptococcus* family as a cause, where sigmoid resection and antibiotics were used in tandem.^17^ Our case had a single abscess, but multiple can arise as well. In cases with large abscesses over 7.3 cm, surgical drainage is preferred to decrease the risk of spontaneous rupture. Antibiotics and drainage were also utilized.^18^ In one case, the culture did not specify *Streptococcus intermedius* but instead showed a polymicrobial mixture with *Candida* as well, the reason being unknown to the authors.^11^ Compared to these reports, our case is notable for the septic presentation of the patient, a single and large abscess, and initial thoughts of an amoebic origin.

The presentation of PLAs is nonspecific and requires a high degree of clinical suspicion. The classical presentation involves fevers, right upper quadrant pain, and malaise.^19^ Other symptoms may include rigors, nausea and vomiting, or cough due to diaphragmatic irritation. Physical exam findings may include right upper quadrant tenderness, jaundice, and hepatomegaly.^19^ Most PLAs are of biliary origin, and laboratory findings often reflect hepatobiliary patterns, including leukocytosis, hyperbilirubinemia, hypoalbuminemia, elevated alkaline phosphatase, or elevated aminotransferases.^11,19^ Our patient presented with signs of a PLA, but his lack of history involving biliary tract disease and recent diverticulitis pointed toward a unique etiology of his abscess.

Treatment for PLAs is usually managed by intravenous antibiotics with broad coverage, including anaerobes. If abscesses are greater than 5 cm, either interventional radiology or CT-guided drainage is needed to control the source. If the abscess has multiple loculations, surgery is beneficial to break down the loculations for overall complete drainage. Ultrasound is typically the mode of imaging; however, a CT scan has a higher specificity in detecting smaller abscesses. Early intervention is key to preventing unnecessary complications, including rupture, sepsis, or pneumonia, the latter being especially prevalent in cases of seeding by *Klebsiella* species.^20^ This case highlights an unusual acute sigmoid diverticulitis case resulting in a liver abscess and showcases the importance of early recognition to reduce the risk of complications.

## Conclusion

PLAs are an uncommon but potentially life-threatening complication of diverticulitis, particularly when arising from portal venous spread. This case highlights the importance of maintaining a high index of suspicion in patients with diverticulitis who present with systemic symptoms and abnormal liver tests. Recognition of these rare etiologies can facilitate earlier intervention, reduce morbidity, and improve overall prognosis in affected patients.
